# Age-related changes in visual search: manipulation of colour cues based on cone contrast and opponent modulation space

**DOI:** 10.1038/s41598-020-78303-4

**Published:** 2020-12-07

**Authors:** Shuto Tamura, Keiko Sato

**Affiliations:** 1 Graduate School of Engineering, Kagawa University, 2217-20 Hayashi-cho, Takamatsu, 761-0396 Japan; 2 Faculty of Engineering and Design, Kagawa University, 2217-20 Hayashi-cho, Takamatsu, 761-0396 Japan

**Keywords:** Colour vision, Human behaviour

## Abstract

Reduced retinal illuminance affects colour perception in older adults, and studies show that they exhibit deficiencies in yellow-blue (YB) discrimination. However, the influence of colour cues on the visual attention in older individuals remains unclarified. Visual attention refers to the cognitive model by which we prioritise regions within the visual space and selectively process information. The present study aimed to explore the effect of colour on visual search performance in older observers. In our experiment, younger observers wearing glasses with a filter that simulated the spectral transmittance of the aging human lens and older observers performed two types of search tasks, feature search (FS) and conjunction search (CS), under three colour conditions: red-green, YB, and luminance. Targets and distractors were designed on the basis of the Derrington–Krauskopf–Lennie colour representation. In FS tasks, reaction times changed according to colour in all groups, especially under the YB condition, regardless of the presence or absence of distractors. In CS tasks with distractors, older participants and younger participants wearing glasses showed slower responses under chromatic conditions than under the achromatic condition. These results provide preliminary evidence that, for older observers, visual search performance may be affected by impairments in chromatic colour discrimination.

## Introduction

Visual attention refers to the cognitive operations that selectively collect and process critical visual information, and visual search tasks are vital tools for investigating visual attention in humans. In such tasks, participants usually confirm the appearance of a specific target as quickly as possible. The tasks can be manipulated by varying the number of distractors presented with the target. The time required to detect the target (reaction time, RT)^1,2^ or the accuracy in detecting the target when the stimuli appear briefly^3,4^ can be measured and further analysed. Generally, RT and accuracy in visual search are the crucial performance criteria to investigate how covert attention selects and controls important information.

Many studies related to visual attention have been executed on the basis of the feature integration theory (FIT): proposed in the 1980s^5^, this theory presents visual attention as a sequential process strategy. The FIT argues that visual characteristics such as colour and orientation are initially parsed into separate maps in the first stage, and that information from each map is merged in the second stage. This integration of individual characteristics requires the creation of a master map of locations, which indicates what combinations of visual characteristics coexist at each location on the map. The FIT explains the dichotomy between the parallel-like functions of feature searching (FS) and the serial-like functions of conjunction searching (CS). In FS, a target is defined by a unique visual feature (for example, colour, shape, orientation, or size). Thus, the number of distractors has little noticeable effect on target search performance. On the other hand, in CS, the target shares visual characteristics with the distractors and is designed by the combination of two attributes, resulting in a steep RT/CS slope. This is because attention to each item for feature integration is required until the target is identified. Thus, search performance in CS tasks is largely influenced by the number of distractors^5^. However, FIT assumes the absence of the bottom-up factor in the CS condition. In counterpoint to the FIT, the guided search theory posits that attentional location is determined by an interaction of bottom-up stimulus characteristics with top-down strategic processes^6–8^. The bottom-up factor of stimulus indicates the featural dissimilarity of the information at a given location with information at all other locations, while top-down processing refers to the modulation that increases the relative activation of locations containing task-relevant stimulus properties. In visual search, attention will be directed to the item with the highest priority.

Older adults are often slower and less accurate than are younger adults in performing visual-search tasks, suggesting an age-related decline in attentional functioning^9,10^. The slope of RT and display-size function is typically higher for older adults than for younger adults^11,12^. When the visual target is a featural singleton, however, older adults typically exhibit independence between RT and display size, indicating a highly efficient search despite slower overall RT^13,14^. Because target selection is less effortful, “pop-out” occurs due to salient differences between the features of the target and the distractors^9^. On the other hand, there is evidence that an age-related decline occurs specifically in top-down attentional guidance; e.g., research on the effect of age on top-down guidance in a CS task suggests that the age-related decline in search performance is particularly robust^13,15–17^. To summarize, older adults typically feature a slower RT and less accuracy than do younger adults in difficult tasks that rely heavily on visual search and discrimination^10^. Furthermore, older adults experience difficulty in interpreting the cue and setting search parameters when the target varies across tasks^18^.

Visual function declines only slightly or not at all until the age of 50–60 years, after which the decline in visual function rapidly accelerates^19^. The age-induced decline in colour perception specifically is due to reductions in retinal illuminance. Concomitant changes in light transmission through the ocular media limit the amount of light delivered to the cone cells. These developments are exacerbated by the densification and yellowing of the lens, which reduce the transmission of short-wavelength light^20–22^.

The human retina features three types, each of which is sensitive to a different spectrum: short (S)-, medium (M)-, and long (L)-wavelength lights. The human trichromatic mechanism is composed of the cone-contrast system and the opponent modulation system, which yield three pairs of mutually exclusive opponent categories: red-green (L − M), yellow-blue (S − (L + M)), and white-black (L + M). Colour perception in older adults has been investigated by assessing colour discrimination. Knoblauch et al.^23^ suggested that older observers are less able than are younger observers to discriminate colours on the yellow-blue opponent axis. Furthermore, yellow-blue sensitivity could be poorer than red-green sensitivity in older adults over the age of 40 years^24^. These results can be attributed to receptor (and post-receptor) dysfunctions linked to age-related changes in the neurons required to discriminate colour. Moreover, greater reductions in short-wavelength light transmission occurs with increased age, resulting in an increase in yellow-blue discrimination threshold^20^.

Colour also provides top-down guidance to locate items in conjunction search tasks^8^. Hence, in visual search tasks in which the target and the distractors have the same shape but are coloured differently—especially in yellow-blue opponent colours—the search performance of older observers may be worse than that of their younger counterparts. In short, older adults may show declines in bottom-up visual processing at the sensory level. However, in search tasks that require the yellow-blue discrimination, whether bottom–up processes may be insulated from age-related decline, or whether there is any change specifically associated with top-down attentional guidance, is less clear.

**Figure 1:**
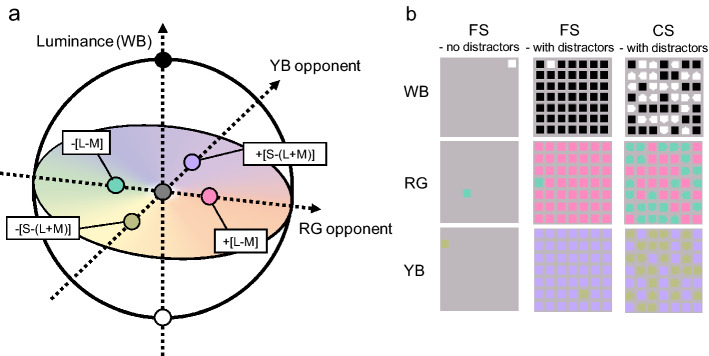
(**a**) DKL colour space, (**b**) sample displays in each condition. *CS* conjunction search, *DKL* Derrington–Krauskopf–Lennie, *FS* feature search, *L* long-wavelength, *M* middle-wavelength, *RG* red-green, *S* short-wavelength, *YB* yellow-blue.

The present study aimed to explore whether colour cues influence visual attention among older subjects as gauged by a visual search task. Visual colour stimuli were defined on the basis of Derrington–Krauskopf–Lennie (DKL) colour space^25^ to investigate the effect of colour based on the cone-contrast and opponent modulation systems. The colour attributes of the target and distractors were composed of the three channels: the red-green opponent (RG), yellow-blue opponent (YB), and white-black (WB) (Fig. 1a). Twenty-four younger observers, half of whom wore glasses with filters simulating the spectral transmittance of the aging human lens, while the other half did not, and 12 older observers underwent visual search testing involving both FS and CS, with and without distractors; hence, the present study elaborates on a prior investigation that reported an effect of colour on search performance among older individuals using only the FS task^26^. For tasks without distractors in the current study, an item was presented at random locations on the display, and the observers responded whether it was the target or not. For tasks with distractors, we added 49 items to the display, and the participants were instructed to judge whether a given target appeared on the display or not. In the FS condition, the target was differentiated from the distractor by a single colour feature (RG, YB, and WB; Fig. 1b). In the CS condition, the target differed from the distractor based on one of the aforementioned colour and shape features (Fig. 1b). RTs and accuracy in detecting targets were measured, and the search performance for all three groups were analysed according to colour conditions.

## Results

To compare the search performance of the age groups, we performed separate three-way analyses of variance (ANOVA) of raw RT, z-transformed RT (zRT) and the number of errors under the FS and CS conditions with the within-subject factors of task (i.e., no-distractor and distractor), colour condition (i.e., WB, RG, and YB), and the between-subject factor of age group (i.e., younger, younger with glasses, and older). Since the primary aim of this study was to examine differences between age groups, only significant interaction effects involving group differences (i.e., task × age group, colour × age group, or task × colour × age group) were evaluated and are discussed here. Therefore, main effects of task and colour, as well as the task × colour interaction are not discussed.

### Raw RT

**Figure 2:**
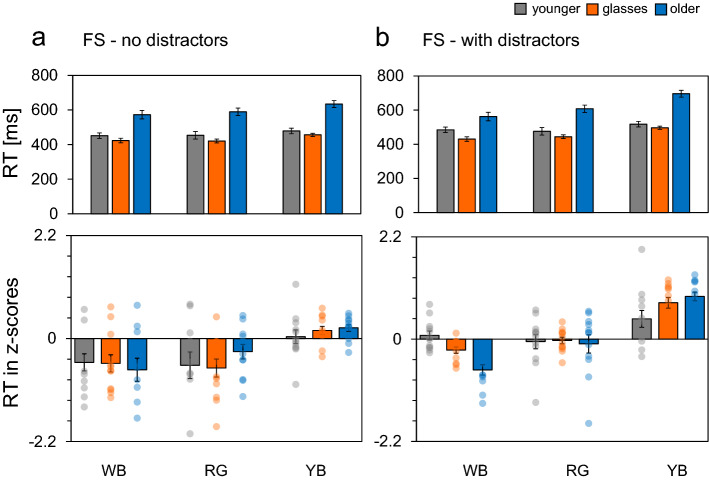
Mean RTs and z-transformed RT for tasks without (**a**) and with distractors (**b**) under the FS condition for younger, younger-with-glasses, and older observers. Error bars indicate the standard error. *RT* reaction time, *FS* feature search, *WB* luminance, *RG* red-green, *YB* yellow-blue.

For the FS tasks, we observed no significant three-way interaction (task × colour × age), and observed significant interaction between colour and age group (*F*(4, 65.95) = 5.18, *p* = 0.001, ${{\eta }_{G}}^{2}$ = 0.039). An analysis of simple effects on the interaction revealed an effect of age for all colour conditions (WB: *F*(2, 33) = 23.8, *p* < 0.001, ${{\eta }_{G}}^{2}$ = 0.539; RG: *F*(2, 33) = 41.6, *p* < 0.001, ${{\eta }_{G}}^{2}$ = 0.648; YB: *F*(2, 33) = 41.6, *p* < 0.001, ${{\eta }_{G}}^{2}$ = 0.648), and an effect of colour for all age groups was observed (younger: *F*(1.99, 21.9) = 5.48, *p* = 0.012, ${{\eta }_{G}}^{2}$ = 0.052; younger with glasses: *F*(1.96, 21.55) = 24.5, *p* < 0.001, ${{\eta }_{G}}^{2}$ = 0.288; older: *F*(1.95, 21.49) = 31.0, *p* < 0.001, ${{\eta }_{G}}^{2}$ = 0.267). Pairwise multiple comparison tests of colour conditions revealed responses under the YB condition to be significantly slower than under the WB (younger: *p* = 0.038; younger with glasses: *p* < 0.001; older: *p* < 0.001) and RG conditions (younger: *p* = 0.038; younger with glasses: *p* < 0.001; older: *p* < 0.001). For older group, responses to RG stimuli were slower than to WB stimuli (*p* = 0.041). The mean RTs for the tasks with and without distractors are shown in the upper panels of Fig. 2a,b, respectively.

**Figure 3:**
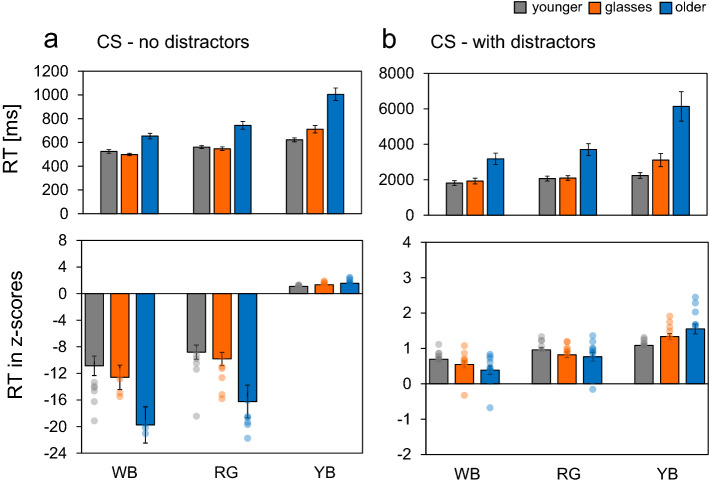
Mean RTs and z-transformed RT for tasks without (**a**) and with distractors (**b**) under the CS condition for younger, younger-with-glasses, and older observers. Error bars indicate the standard error. *RT* reaction time, *CS* conjunction search, *WB* luminance, *RG* red-green, *YB* yellow-blue.

For CS tasks, we observed a significant three-way interaction between task, colour and age group (*F*(2.31, 38.1) = 5.81, *p* = 0.005, ${{\eta }_{G}}^{2}$ = 0.061). To compare the search performance of the age group under each task type, we performed separate two-way ANOVA. For tasks without distractors, we observed significant interaction between colour and age group (*F*(2.75, 45.38) = 9.80, *p* < 0.001, ${{\eta }_{G}}^{2}$ = 0.204). A simple effects analysis for this interaction revealed an effect of age for each colour condition (WB: *F*(2, 33) = 23.6, *p* < 0.001, ${{\eta }_{G}}^{2}$ = 0.589; RG: *F*(2, 33) = 25.1, *p* < 0.001, ${{\eta }_{G}}^{2}$ = 0.603; YB: *F*(2, 33) = 29.7, *p* < 0.001, ${{\eta }_{G}}^{2}$ = 0.643), and an effect of colour at each age (younger: *F*(1.87, 20.52) = 19.9, *p* < 0.001, ${{\eta }_{G}}^{2}$ = 0.408; younger with glasses: *F*(1.15, 12.6) = 42.7, *p* < 0.001, ${{\eta }_{G}}^{2}$ = 0.634; older: *F*(1.38, 15.16) = 34.8, *p* < 0.001, ${{\eta }_{G}}^{2}$ = 0.578). Pairwise multiple comparison tests for colour conditions revealed responses under the YB condition to be significantly slower than under the WB (younger: *p* < 0.001; younger with glasses: *p* < 0.001; older: *p* < 0.001) and RG conditions (younger: *p* = 0.002; younger with glasses: *p* < 0.001; older: *p* < 0.001). Furthermore, the RG condition induced slower responses that did the WB condition (younger: *p* = 0.023, younger with glasses: *p* < 0.001; older: *p* = 0.005). For tasks with distractors, we observed significant interactions between age and colour (*F*(2.28, 37.6) = 6.51, *p* = 0.003, ${{\eta }_{G}}^{2}$ = 0.138). For this interaction, a simple effects analysis revealed an effect of age for each colour (WB: *F*(2, 33) = 11.5, *p* < 0.001, ${{\eta }_{G}}^{2}$ = 0.412; RG: *F*(2, 33) = 17.9, *p* < 0.001, ${{\eta }_{G}}^{2}$ = 0.521; YB: *F*(2, 33) = 14.8, *p* < 0.001, ${{\eta }_{G}}^{2}$ = 0.472), and an effect of colour at each age (younger: *F*(1.42, 15.66) = 13.9, *p* < 0.001,${{\eta }_{G}}^{2}$ = 0.120; younger with glasses: *F*(1.05, 11.6) = 13.0, *p* = 0.004, ${{\eta }_{G}}^{2}$ = 0.291; older: *F*(1.12, 12.36) = 12.6, *p* = 0.003, ${{\eta }_{G}}^{2}$ = 0.333). Pairwise multiple comparison tests for colour conditions revealed that YB stimuli yielded slower responses than did the WB (younger: *p* < 0.001; younger with glasses: *p* = 0.006; older: *p* = 0.008) and RG stimuli (younger: *p* = 0.014; younger with glasses: *p* = 0.009; older: *p* = 0.008). For all ages, responses to RG stimuli were slower than to WB stimuli (younger: *p* = 0.032; younger with glasses: *p* = 0.020; older: *p* = 0.033). The mean RTs for tasks with and without distractors are shown in the upper panels of Figs. 3a,b, respectively.

### Z-transformed RT

Z-transformation controls for individual differences in the baseline RT^27^ and confirms whether the differences between younger and older observers reflect a qualitative difference or simply age-related slowing. Referring to this analysis, the overall mean of each individual was subtracted from the mean of each condition; the difference was then divided by the standard deviation of the condition means. zRT was calculated separately for the FS and CS conditions and followed by the performance of an ANOVA.

For the FS tasks, we observed no significant three-way interaction (task × colour × age), and observed a significant interaction between colour and age group (*F*(3.47, 57.3) = 3.18, *p* = 0.025, ${{\eta }_{G}}^{2}$ = 0.065). An analysis of simple effects on the interaction revealed an effect of age for the WB and YB colour conditions (WB: *F*(2, 33) = 4.25, *p* = 0.023, ${{\eta }_{G}}^{2}$ = 0.108; YB: *F*(2, 33) = 7.04, *p* = 0.003, ${{\eta }_{G}}^{2}$ = 0.110), and an effect of colour for all age groups was observed (younger: *F*(1.31, 14.36) = 5.91, *p* = 0.022, ${{\eta }_{G}}^{2}$ = 0.135; younger with glasses: *F*(1.87, 20.56) = 21.3, *p* < 0.001, ${{\eta }_{G}}^{2}$ = 0.454; older: *F*(1.48, 16.3) = 25.2, *p* < 0.001, ${{\eta }_{G}}^{2}$ = 0.486). Pairwise multiple comparison tests of colour conditions revealed responses under the YB condition to be significantly slower than under the WB (younger: *p* = 0.004; younger with glasses: *p* < 0.001; older: *p* < 0.001) and RG conditions (younger: *p* = 0.007; younger with glasses: *p* < 0.001; older: *p* < 0.001). In the older group, responses to RG stimuli were slower than to WB stimuli (*p* = 0.054). The mean zRTs for the tasks with and without distractors are shown in the lower panels of Fig. 2a,b, respectively.

For CS tasks, we observed a significant three-way interaction between task, colour, and age group (*F*(3.45, 56.94) = 3.34, *p* = 0.021, ${{\eta }_{G}}^{2}$ = 0.023). To compare the search performance of the age group under each task type, we performed a separate two-way ANOVA. For tasks without distractors, we observed a significant interaction between age and colour (*F*(3.54, 58.45) = 3.91, *p* = 0.009, ${{\eta }_{G}}^{2}$ = 0.056). A simple effects analysis for this interaction revealed an effect of age for the WB and RG conditions (WB: *F*(2, 33) = 5.15, *p* = 0.011, ${{\eta }_{G}}^{2}$ = 0.238; RG: *F*(2, 33) = 5.91, *p* = 0.006, ${{\eta }_{G}}^{2}$ = 0.264), and an effect of colour at each age (younger: *F*(1.84, 20.26) = 3.88, *p* = 0.040, ${{\eta }_{G}}^{2}$ = 0.086; younger with glasses: *F*(1.59, 17.45) = 14.5, *p* < 0.001, ${{\eta }_{G}}^{2}$ = 0.318; older: *F*(1.77, 19.45) = 18.3, *p* < 0.001, ${{\eta }_{G}}^{2}$ = 0.261). Pairwise multiple comparison tests for colour conditions revealed that the YB condition induced slower responses than did the WB (younger with glasses: *p* = 0.001; older: *p* < 0.001) and RG conditions (younger with glasses: *p* = 0.001; older: *p* < 0.001). Responses in the younger group did not change among the colour conditions. For tasks with distractors, we observed significant interactions between age and colour (*F*(3.35, 55.26) = 4.50, *p* = 0.005, ${{\eta }_{G}}^{2}$ = 0.165). For this interaction, a simple effects analysis revealed an effect of age for the YB condition (*F*(2, 33) = 5.63, *p* = 0.008, ${{\eta }_{G}}^{2}$ = 0.254), and an effect of colour at each age (younger: *F*(1.39, 15.27) = 10.9, *p* = 0.003, ${{\eta }_{G}}^{2}$ = 0.443; younger with glasses: *F*(1.42, 15.63) = 19.9, *p* < 0.001; ${{\eta }_{G}}^{2}$ = 0.557; older: *F*(1.45, 15.9) = 20.0, *p* < 0.001, ${{\eta }_{G}}^{2}$ = 0.568). Pairwise multiple comparison tests for colour conditions revealed the YB condition to be slower than the WB (younger: *p* = 0.001; younger with glasses: *p* = 0.001; older: *p* < 0.001) and RG conditions (younger with glasses: *p* = 0.002; older: *p* = 0.005). Responses under the RG condition were slower than under the WB condition (younger: *p* = 0.034; younger with glasses: *p* = 0.008; older: *p* = 0.008). The mean zRTs for the tasks with and without distractors are shown in the lower panels of Fig. 3a,b, respectively.

### Errors

**Figure 4:**
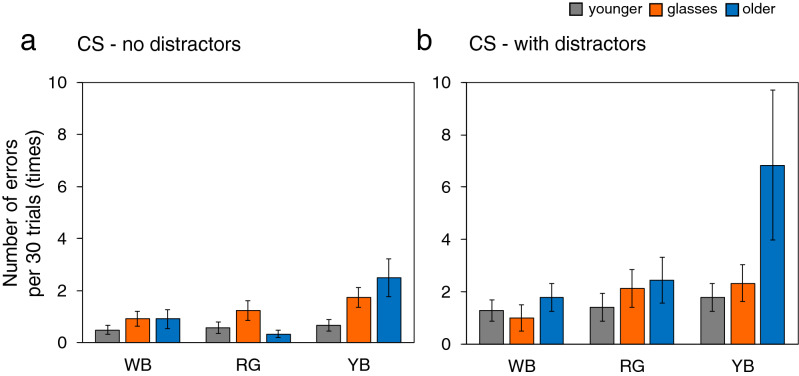
Number of errors per 30 trials without (**a**) and with distractors (**b**) under the CS condition for younger, younger-with-glasses, and older participants. Error bars indicate the standard error. *RT* reaction time, *CS* conjunction search, *WB* luminance, *RG* red-green, *YB* yellow-blue.

Similarly, we performed a mixed ANOVA using task, colour and age as factors to examine the number of errors made in the FS and CS tasks. For the FS tasks, we observed no significant interactions for either task type. For CS tasks, we observed a significant three-way interaction between task, colour and age (*F*(2.48, 40.91) = 7.02, *p* = 0.001, ${{\eta }_{G}}^{2}$ = 0.072). To compare the search performance of the age group under each task type, we performed separate two-way ANOVA. For the tasks without distractors, we observed significant interactions between age and colour (*F*(3.16, 52.11) = 3.52, *p* = 0.020, ${{\eta }_{G}}^{2}$ = 0.092). A simple effects analysis following a significant interaction between age and colour revealed an effect of age at YB (*F*(2, 33) = 3.66, *p* = 0.037, ${{\eta }_{G}}^{2}$ = 0.182) and an effect of colour among younger participants with glasses (*F*(1.64, 18.05) = 4.18, *p* = 0.039, ${{\eta }_{G}}^{2}$ = 0.082) and older participants (*F*(1.32, 14.51) = 6.75, *p* = 0.015, ${{\eta }_{G}}^{2}$ = 0.258). However, pairwise multiple comparison tests for colour conditions revealed no significant differences between colour conditions for either younger observes with glasses or older observers. For tasks with distractors, we found a significant interaction between age and colour (*F*(2.37, 39.05) = 8.78, *p* < 0.001, ${{\eta }_{G}}^{2}$ = 0.200). A simple effects analysis revealed effects of age at YB (*F*(2, 33) = 10.23, *p* < 0.001, ${{\eta }_{G}}^{2}$ = 0.383) and an effect of colour among younger observers with glasses (*F*(1.71, 18.83) = 12.00, *p* < 0.001, ${{\eta }_{G}}^{2}$ = 0.228) and older participants (*F*(1.04, 11.47) = 12.93, *p* = 0.004, ${{\eta }_{G}}^{2}$ = 0.371). Pairwise multiple comparison tests for colour conditions revealed that both younger participants with glasses and older observers made more errors under the YB than under the WB condition (younger with glasses: *p* = 0.007; older: *p* = 0.012) and more errors under the RG condition than under the WB condition (younger with glasses: *p* = 0.007; older: *p* = 0.043). Only older observes made more errors under the YB condition than under the RG condition (*p* = 0.012). The mean number of errors per 30 trials for CS tasks with and without distractors is shown in Fig. 4a,b, respectively.

### Additional experiment using non-equivalent luminance contrast

We performed an additional experiment for younger and older participants to investigate search performance under the chromatic colour conditions with non-equivalent luminance contrast (only CS tasks). Under the non-equivalent luminance condition, we observed no significant interactions between age and colour for either task type (see Supplementary Fig. S2). We further compared RTs between the additional experiment (black background: high contrast) and the first experiment (grey background: low contrast) by three-way ANOVA using age, background type, and colour as factors. For tasks without distractors, there was a significant interaction between age, background type, and colour condition (*F*(1, 44) = 12.64, *p* < 0.001, ${{\eta }_{G}}^{2}$ = 0.063). Among younger observers, there were no differences between background types under either colour condition; however, among older observers, a significant interaction was observed (*F*(1, 22) = 23.69, *p* < 0.001, ${{\eta }_{G}}^{2}$ = 0.193). There was a significant difference between background types only under the YB condition (see Supplementary Fig. S3).

For tasks including distractors, we observed a significant interaction between age, background type, and colour (*F*(1, 44) = 7.15, *p* = 0.011, ${{\eta }_{G}}^{2}$ = 0.045). For both younger and older observers, there were significant interactions between background type and colour condition (younger: *F*(1, 22) = 4.60, *p* = 0.043, ${{\eta }_{G}}^{2}$ = 0.011; older: *F*(1, 22) = 7.92, *p* = 0.006, ${{\eta }_{G}}^{2}$ = 0.112). Under the YB condition, RTs were significantly slower for the grey background than for the black background among older observers, but not among younger observers (see Supplementary Fig. S4).

## Discussion

The present study aimed to explore the effect of colour on visual search performance among older individuals. For FS tasks, we observed no significant interaction between age, task type (with and without distractor) and colour, but a significant interaction between age and colour was noted. Regardless of the presence or absence of distractors, RTs under the YB condition were significantly slower than under the WB and RG conditions among younger observers and younger observers with glasses whose lenses emulated the aging human lens. A significant difference in RTs to WB and RG stimuli was only found for older observers. RTs in older observers were slowest under the YB condition and fastest under the WB condition.

When the target and the distractor dimensions differed only in colour, especially when colours in the S − (L + M) direction (YB colours) were used, we observed a significant difference in RTs among chromatic colour conditions (RG and YB) for older participants and younger participants wearing the glasses as predicted. However, we observed a significant difference in RTs between the RG and YB conditions, even among younger participants. Previous research has associated the S-opponent system with a longer response latency than that of the L/M-opponent system^28,29^. Smithson and Mollon^30^ confirmed this finding psychophysically by observing a 20–30 ms-difference between the L/M-opponent and S-opponent systems. Exploring the effect of chromaticity on RT for a variety of stimulus conditions, O’Donell et al.^31^ reported that RTs were strongly influenced by stimulus size when the chromatic stimulus is modulated along the S − (L + M) pathway. Furthermore, Lindsey et al.^32^ suggested that visual searching for reddish targets is much easier than for purplish targets, which induce greater excitation of the S cones. These studies suggest the differences between the temporal responsiveness of the L − M or S − (L + M) opponent mechanism.

RTs to RG stimuli were significantly slower than WB stimuli for older observers. This result can be attributed to the fact that the chromatic sensitivities are not uniform throughout the lifespan^33^. Both the RG and YB discrimination thresholds gradually increase across the life span^20,34^. In our experiment, to ascertain if the differences between younger and older observers reflect the reduction of transmittance of short-wavelength light, we used glasses with filters simulating the spectral transmittance of an aging human lens^35^. Previous research using these glasses has indicated that the yellowing of the human lens strongly influences reaction time for colour targets^36^. However, the RTs of younger participants with aging filters did not quantitatively coincide with those of the elderly participants. RTs are markedly slower for older participants than for younger participant wearing glasses, and is particular susceptible to the influence of colour^36^. The current results also indicated that search performance in the FS task was more affected by colour conditions in older participants than in younger participants wearing glasses.

For older participants, RTs were slower in the CS task than in the FS task. Trick and Enns^37^ reported that older observers responded more slowly than younger observers under the CS condition, suggesting that older observers may be less capable than younger observers to voluntarily shift their attention between items. Scialfa and Joffe^16^ indicated that age-related deficits in RTs were greatest in CS tasks and target-absent trials. Furthermore, Muller-Obhring^38^ mentioned that while older adults can capitalize on early parallel stages of visual information processing, age-related limitations arise at later serial processing stages; this conclusion further implies a requirement for self-guided selective attention and executive control. In accordance with these previous research studies on visual search for the conjunction of two features^13,15–17,37^, the results of our study suggest that the age-related decline in CS task performance is particularly robust.

For CS tasks with and without distractors, we observed significant interactions between age and colour for raw RTs. For all groups, RTs were slowest under the YB condition and fastest under the WB condition. However, the differences among colour conditions were greater for younger participants with glasses than for younger observers, and were much greater for older observers than for their younger counterparts. Among younger observers, RTs in the CS task with distractors differed between the RG and YB conditions by approximately 170 ms; this difference increased to 1000 and 2400 ms among younger observers with glasses and older observers, respectively.

For z-transformed RTs that controlled for individual differences, we observed significant interactions between age and colour under the task without distractors. For the younger group, RTs did not change among the colour conditions, however RTs in younger participants with glasses and older group were slowest under the YB condition and fastest under the WB and RG conditions. In the tasks with distractors, RTs in younger participants were slower for chromatic colour stimuli (RG and YB) than for achromatic stimuli (WB). Younger participants with glasses and the older group showed the slowest responses under the YB condition, followed by the RG, and WB conditions.

Under the CS condition, the target presented was a square in the target colour, and the distractor was either a pentagon in the target colour or a square in a non-target colour (Fig. 1b). In this task, a contrast in luminance between the background and stimuli is assumed to be an effective cue for detecting contours. Employing a visual search task that used shape as a feature under conditions of high- and low-luminance contrast between the background and stimuli, Takahashi et al.^39^ reported RTs to be higher under the low-contrast condition than under the high-contrast condition. In addition, Tollner et al.^40^ adopted a visual search task that required participants to respond to the orientation of a line within the target under three contrast conditions (low, middle, and high); their study found that RTs increased as contrast decreased. Furthermore, using a dual task paradigm in which the observer was required to detect peripheral test stimuli while performing an attention task, Uchikawa et al.^41^ reported that the chromatic RG and YB directions required greater attentional resources than the achromatic WB direction; as in the present study, Uchikawa et al.^41^ created peripheral stimuli based on the L + M, L − M, and S − (L + M) axes. In the present experiment the target colours under the chromatic colour conditions had the same luminance as the background (equivalent luminance contrast). This method may have rendered the detection of target’s contour more difficult under the chromatic condition than under the achromatic condition. This supposition prompted us to perform additional experiments involving both younger and older participants to investigate search performance under the chromatic colour conditions with non-equivalent luminance contrast: we only observed significant differences in RTs to RG and YB stimuli among older participants, suggesting that older individuals experienced difficulty in detecting the target under the YB condition due to a decreased ability to differentiate two colours on the S − (L + M) axis.

Under the CS task, younger participants with glasses showed slower responses under the YB condition than under the RG condition, which was similar to the results of the older group. The lens filter, which simulated the yellowing of the aging human lens, modifies the colour difference between the colour target and the background on the retina^36^. This modification lengthened the RTs of younger observers with glasses. RTs under the YB condition in the CS tasks were markedly slower for older observers due to the age-related decline in search performance. This was especially apparent when visual selection rendered the discernment of the target from the surrounding distractors more difficult^10^. Our stimuli in the chromatic condition, as aforementioned, were specified at equivalent luminance. Hence, participants needed to detect the target shape using only chromatic information. In the chromatic CS tasks, more attentional resources were required to discriminate the edge of the target from the background colour, resulting in decreased performance, particularly under the YB condition (i.e. for S-cone isolating stimuli).

Concerning the number of errors, we observed significant differences in tasks containing distractors among colour conditions in the younger observes with glasses and the older group. The latter had the highest number of errors involving large inter-subject variability under the YB condition, followed by the RG and WB conditions in that order. By contrast, among younger observers wearing glasses, the number of errors did not differ between the RG and YB conditions. This may have been due to changes in search strategy across age. Coyne et al.^42^ indicated that older observers are more likely to adopt an impulsive search strategy than are younger observers. The difference in the number of errors between the younger observers wearing glasses and their older counterparts may thus be attributed to the impulsivity and individual differences among the latter.

We considered the search performance in the CS task for older observers both in terms of top-down and bottom-up attentional processing. Since Madden et al.^43^ reported that younger adults benefit more from distractor homogeneity in CS tasks and achieve faster RTs than do older adults, it has been assumed that older adults experience difficulties in both perceptual grouping (bottom-up processes) and the inhibition of distractors (top-down processes). Most forms of visual search represent the combined influence of top-down and bottom-up attentional processing. Attentional guidance can be either top-down (i.e., can depend primarily on the observer's goals and knowledge of the task structure) or bottom-up (i.e., can be relatively more determined by the local properties of the visual display)^44^. Top-down attention is essential in complex search tasks such as the CS condition. In contrast, when the target differs from the distractors in a unique feature, such as in the FS condition, the target visually pops out of the display visually in a bottom-up (stimulus-driven) manner. However, Anderson et al.^45^ discussed search performance in the CS condition in terms of the bottom-up process; they argued that the bottom-up parsing of visual information into sub-groups is affected by the discriminability between the target and the distractors within each dimension defining the target. In particular, they demonstrated that differences in the colour of the target and distractors are critical. Thus, the presence of a colour cue may enhance the bottom-up process whereby elements of visual information are parsed into sub-groups. Furthermore, Anderson et al.^46^ provided evidence for a two-stage framework for the top-down guidance of attention under the CS condition and proposed that visual displays are initially parsed into colour feature-based groups (i.e., a process modulated by the presence of an initial cue to the colour of target). Subsequently, searching is directed to other features of the colour-based groups. Hence, cues to other features of the target are only effective at the second stage (i.e., the search process is re-configured in the CS condition based on the presence of top-down cues). This two-stage framework supports the notion that, in CS tasks with distractors, observers initially carry out bottom-up parsing of the visual information into colour-based groups^46^. This process is affected by the discriminability between the target and the distractors. Subsequent searching is directed to the shape feature of the colour-based groups. Hence, for older observers, the search process in the YB condition was affected by deficiencies in chromatic colour discrimination (i.e., the colour-based group could not be adequately constructed). Participants were consequently required to rely on shape features in the next stage of searching. Search performance was further affected by low luminance contrast, which confounded the detection of the edges of a shape in the present study. However, we should note that distinguishing a deficit in executive control from a more basic mechanism, such as that of perceptual speed, is difficult.

The present study is subjected to some limitations. First, although the older participants had normal colour vision, the older participants in this study did not undergo medical evaluations for visual field, peripheral colour vision, or light scatter, nor were they screened for eye diseases with ophthalmological assessment. Hence, we cannot differentiate the effects of eye diseases that may affect the retina from the gradual decline in vision ascribed to normal aging^20^. The prevalence of many ocular diseases increases substantially with age: e.g., age-related macular degeneration, glaucoma, diabetic retinopathy, optic neuropathies, etc. Vision-limiting media opacities such as age-related cataracts, irregularities of the cornea or sclera are also more prevalent among older populations^19^. Furthermore, intraocular light scattering induced by age-related cataracts results in a veiling illuminance superimposed upon the retinal image and a concomitant reduction of retinal contrast^47,48^. The ocular diseases, characteristic of older adults, generate variability in colour perception. For instance, Paramei and Oakley^49^ suggested that there is greater variability in colour detection thresholds with an increase in scatter among individuals over the age of 60 years. Such distribution issues in the colour perception of older observers may have influenced the current results. This limitation may have been exacerbated by our relatively limited sample. Future studies should take these issues into consideration by emphasizing the recruitment of individuals who represent the average status of older populations without ocular disease. Furthermore, Berine et al. indicated that the short wavelength-sensitive function in the periphery declines with increasing age from the detection tasks^50^. In order to observe the effects of declined visual acuity and short-wavelength sensitive function in peripheral vision, ophthalmological assessment for older participants and accurately controlled presentations of stimuli are needed.

The present study may have further been limited by the fact that the stimuli used in the RG and YB conditions were defined with a specific contrast in the DKL colour space, and not across the full range of colour contrast. It is thus possible that the presently reported results depend on contrast level. Confirmation of this possibility warrants further investigation. However, the results of the present study provide preliminary evidence that, for older observers, visual search performance is affected by impairments in chromatic colour discrimination, especially of the S-cone isolating stimuli.

## Methods

### Participants

The present study included 12 younger observers (mean age ± SD 21.9 ± 0.79 years; age range 21–23 years), 12 younger observers (mean age ± SD 22.4 ± 1.08 years; age range 21–24 years) who wore glasses with filters simulating the spectral transmittance of the aging human lens, and 12 older observers (mean age 70.7 ± 3.47 years; age range 66–77 years). The additional experiment included 12 younger observers (mean age 22.3 ± 1.23 years; age range 21–25 years) and 12 older observers (69.1 ± 4.38 years; age range 64–79 years). The younger observers were recruited from universities, and the older observers were residents from nearby communities. All participants had normal or corrected-to-normal vision acuity (20/32 or better) assessed by Freiburg Visual Acuity Test^51^, and normal color vision assessed using Ishihara pseudoisochromatic plates^52^. Further, the older participants were also screened with the Mini-mental State Examination (MMSE), and none of them showed early symptoms of dementia. Written informed consent was obtained from all participants, and the experiments was performed in accordance with the Declaration of Helsinki and with approval from the Ethics Committee of Kagawa University (28-005, 01-003). All individuals participated in our experiment voluntarily and received a gift certificate as compensation.

### Apparatus

We conducted the experiments in a dark laboratory room under D65 ceiling lighting. The visual stimuli were presented on a monitor (EIZO, ColorEdge CX 270, 1920 × 1200 resolution), which was calibrated for a white reference point (x = 0.3153, y = 0.3226, Y = 98.21 cd/m^2^) and the Adobe RGB colour gamut. In addition, colour temperature was set to 6500 K, and the gamma value of the monitor was set to 2.2 using a colorimeter (EIZO, EX2). The heads of the participants were fixed using a chin rest, and the viewing distance between the monitor and the observers was fixed at 50 cm. Twelve younger participants were asked to wear glasses (Itoh Optical Industrial Co., Ltd., SeniorView™) with filters simulating the spectral transmittance of the aging human lens^35^. The normalized reflectance of this filter measured by the spectrometer (StellarNet Inc., BLACK-Comet UV–VIS) is illustrated in Supplementary Fig. S5. We controlled the experiment program using MATLAB (version 9.4.0.813654, Mathworks, USA) and Psychtoolbox (version 3.0.14, http://psychtoolbox.org).

### Stimuli

As in our prior study^26^, all colour stimuli were designed based on the DKL colour representation. The DKL colour representation^25,53,54^, an extension of the MacLeod–Boynton chromaticity diagram^55^, is similar to the cone-contrast space, which is based on a model of early visual processing. This model focuses on responses to the background and deviations from the background, reflecting the fact that the DKL colour space represents colour contrasts among stimuli; i.e., colour is defined by modulations along three different cardinal axes. First, the L-, M-, and S-cones are modulated along the achromatic direction such that the contrast to the background is identical (i.e. ΔL/L_b_ = ΔM/M_b_ = ΔS/S_b_, where ΔL, ΔM, and ΔS represent the differences between each stimulus and the background, respectively; and L_b_, M_b_, and S_b_, indicate the L-, M-, and S-cone excitations for the background colour). The second direction expresses a modulation along an RG axis to maintain constant excitation of the S-cones—i.e. ΔS = 0—and vary that of the L and M cones such that their sum remains constant. Thus, this direction is referred to as an RG isoluminant axis^53^. Only the S cone is modulated along the third direction: ΔL and ΔM = 0. Therefore, this direction is referred to as an S-cone isoluminant axis^53^. The two chromatic axes intersect at the grey point and span an isoluminant plane through the grey point. All lights in this plane have the same luminance as defined by the sphotopic luminosity function^56^.

The colour stimuli are often defined in terms of the responses of a set of hypothesised post-receptor mechanisms that are isolated by these cardinal colour modulations^25,53^. The three directions represent two opponent colour mechanisms and an achromatic mechanism (see Fig. 1a). One of the two opponent axes is a reddish–greenish opponent system that considers the weighted difference between the differential L-and M-cone excitations; the other is a yellow-violet opponent system that considers the weighted difference between the differential S-cone and the sum of the differential L- and M-cone excitations. The achromatic axis sums up the weighted differential L- and M- cone signals. These mutually orthogonal directions are referred to as ‘L − M’, ‘S − (L + M)’, and ‘L + M’^25^. In this paper, we defined the two chromatic directions and achromatic direction of our stimuli as follows: red-green axis, ‘L − M’; yellow-violet axis, ‘S − (L + M)’; achromatic axis, ‘L + M’.

To define a colour stimulus based on the DKL colour model, we measured the tristimulus XYZ values of R, G, B, and the white point, which represents the limits of our display, using a luminance and colour metre (KONICA MINOLTA, CS-150). We translated the tristimulus XYZ values into amounts of L-, M-, and S-cone excitation based on the cone fundamentals of Hunt–Pointer–Esteves (normalised to illuminant D65)^57^. ΔL, ΔM, and ΔS were then calculated using the following equations: ΔL = (L_s_ − L_b_)/L_b_, ΔM = (M_s_ − M_b_)/M_b_, and ΔS = (S_s_ − S_b_)/S_b_. The subscript ‘s’ in each equation denotes the stimulus; and subscript ‘b’, the background colour. Lastly, the DKL values were calculated according to the cone-contrast weights defined by Brainard^53^.

The endpoints of the L − M and S − (L + M) axes were determined by the available monitor gamut. Increments and decrements along the L − M axis were 0.21 (+ [L − M]) and − 0.16 (− [L − M]) for the L- and M-cone contrasts, respectively. Positive modulations along the S − (L + M) axis were 0.0 for the L- and M-cone contrasts and 0.89 for the S-cone contrast (+ [S − (L + M)]). Negative modulations were 0.0 for the L- and M-cone contrasts and − 0.43 for the S-cone contrast (− [S − (L + M)]). The CIE 1931 coordinates of the neutral grey background were x = 0.3132 and y = 0.3091, and the luminance value was 49.26 cd/m^2^. Additionally, we used black (Y = 1.135 cd/m^2^) and white (Y = 97.40 cd/m^2^) stimuli modulated along the luminance (L + M) direction. The measured values of the stimuli are shown in Supplementary Table S1.

Furthermore, we performed an additional experiment with the luminance of the background set to 1.135 cd/m^2^. Two chromatic colour conditions (YB and RG conditions) were performed with and without distractors (see Supplementary Fig. S1).

### Procedure

Participants performed FS and CS tasks under the three colour conditions: the YB, in which − [S − (L + M)] vs. + [S − (L + M)] stimuli appeared on the monitor; RG, in which + [L − M] vs. − [L − M] stimuli appeared on the monitor; and WB, in which white vs. black were displayed. In all tasks, the target colour was − [S − (L + M)] in the YB condition, − [L − M] in the RG condition, and white in the WB condition. In FS tasks, the target was defined by colour only, and squares were presented in the target or non-target colour. In CS tasks, the target was defined by colour and shape, and squares in the target colour, squares in the non-target colour, and pentagons in the target colour were presented. In all conditions, the target was a square with the target colour. Each condition included trials with and without distractors. For tasks without distractors, only one item (the target or the distractor) always appeared on the monitor (Fig. 1b). This item was presented anywhere in the area of 16.6° × 16.6°. For the CS and FS tasks with distractors, the target was presented in a random location along with 48 distractors (Fig. 1b).

The participants performed the search task under 12 conditions, which consisted of the search type and the presence or absence of distractors under each colour condition [two search types: FS and CS tasks × two conditions: without distractors and with distractors × three colour conditions: WB, RG, and YB]. The participants performed the FS task and the CS task without distractors followed by the same tasks with distractors. The order of presentation of the colour conditions was counterbalanced across trials. The target was displayed as a square patch, and distractors appeared as square or pentagonal patches of 1.83° × 1.83° in size. Under each condition, a target appeared in half of the trials, and the participants were instructed to determine whether a target was present as quickly and accurately as possible using a keyboard. Each condition included 30 trials without distractors and 60 trials with distractors. Prior to each condition, the participants completed 15 practice trials. A fixation point displayed at a visual angle of 0.69° in diameter appeared for 1000 ms between trials. Furthermore, visual feedback (‘Error!’) for incorrect responses was presented for 2000 ms at the centre of the monitor. Each participant completed all tasks, including the practice trial, within 90 min; the participants were allowed 5-min breaks to lessen the effects of fatigue.

### Statistical analyses

Results are presented as means $\pm$ SEM. All response values lying more than three standard deviations above or below the mean for a given participant were excluded. The distributions were systematically tested for normality before the appropriate tests were applied. Parametric tests (repeated-measures ANOVA, Greenhouse–Geisser’s adjustment to correct for violating the assumption of sphericity with repeated-measures ANOVA, and Shaffer’s modified sequentially rejective procedure for multiple comparisons) were performed using R-open source software, version 3.4.1 (R Development Core Team). A *p*-value of < 0.05 was considered significant.

## Supplementary Information

Supplementary Information.

## Data Availability

All data generated or analysed during this study are included in this published article. The datasets generated during and/or analysed during the current study are available from the corresponding author on reasonable request.
