# Supplementary material for: Age-related changes in visual search: manipulation of colour cues based on cone contrast and opponent modulation space
Source: Sci Rep. 2020 Dec 7;10:21328. doi: 10.1038/s41598-020-78303-4 (PMC7721812; doi:10.1038/s41598-020-78303-4)
Supplement: Supplementary file 1 — Supplementary Information. [file 41598_2020_78303_MOESM1_ESM.docx]

**Age-related changes in visual search: manipulation of colour cues based on cone contrast and opponent modulation space**

**Shuto Tamura^1^, Keiko Sato^2,*^**

^1^Graduate School of Engineering, Kagawa University, 2217-20 Hayashi-cho, Takamatsu 761-0396, Japan

^2^Faculty of Engineering and Design, Kagawa University, 2217-20 Hayashi-cho, Takamatsu 761-0396, Japan

^*^Correspondence and requests for materials should be addressed to K.S. (email: sato.keiko@kagawa-u.ac.jp).


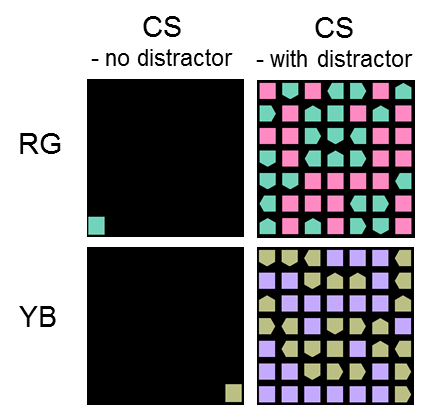


Supplementary Figure S1. Sample displays of additional experiment (black background) under each condition. RG: red-green; YB: yellow-blue; CS: conjunction search.

Supplementary Figure S2. Mean RTs for additional CS task under black background without (a) and with distractors (b) for younger and older observers. Error bars indicate the standard error. RT: reaction time; CS: conjunction search; RG: red-green; YB: yellow-blue.

Supplementary Figure S3. Mean RTs for CS task without distractors for younger (a) and (b) for older observers under the first experiment (grey background) and additional experiment (black background). Error bars indicate the standard error. RT: reaction time; RG: red-green; YB: yellow-blue.

Supplementary Figure S4. Mean RTs for CS task containing distractors for younger (a) and (b) for older observers under the first experiment (grey background) and additional experiment (black background). Error bars indicate the standard error. RT: reaction time; RG: red-green; YB: yellow-blue.

Supplementary Figure S5. Normalized reflectance of glasses with filters simulating the spectral transmittance of the aging human lens [29], which measured by the spectrometer (StellarNet Inc., BLACK-Comet UV-VIS).

Supplementary Table S1. Measured CIE 1931 xy value and the Y value of the stimuli

|  | x | y | Y[cd/m^2^] |
| --- | --- | --- | --- |
| +[S-(L+M)] | 0.2770 | 0.2770 | 49.42 |
| -[S-(L+M)] | 0.3522 | 0.3913 | 49.60 |
| +[L-M] | 0.3991 | 0.2670 | 49.77 |
| -[L-M] | 0.2300 | 0.3537 | 49.08 |
| White | 0.3107 | 0.3140 | 97.40 |
| Black | 0.3029 | 0.3184 | 1.135 |
| Background | 0.3132 | 0.3091 | 49.26 |
